# The roles for innate lymphoid cells in the human immune system

**DOI:** 10.1007/s00281-018-0688-7

**Published:** 2018-06-12

**Authors:** L. Mazzurana, A. Rao, A. Van Acker, J. Mjösberg

**Affiliations:** 1 Center for Infectious Medicine, Department of Medicine, Karolinska Institutet, Stockholm, Sweden; 2 Department of Clinical and Experimental Medicine, Linköping University, Linköping, Sweden

**Keywords:** Innate lymphoid cells (ILCs), Immune regulation, Plasticity, Immune cell interactions

## Abstract

From constituting a novel and obscure cell population, innate lymphoid cells (ILCs) are now accepted as a self-evident part of the immune system, contributing with unique and complementary functions to immunity by production of effector cytokines and interaction with other cell types. In this review, we discuss the redundant and complementary roles of the highly plastic human ILCs and their interaction with other immune cells with the ultimate aim of placing ILCs in a wider context within the human immune system.

## Introduction

Innate lymphoid cells (ILCs) is the collective term for a group of lymphoid cells lacking rearranged antigen-specific receptors [1] of which the natural killer (NK) cells are the most well characterized. However, in addition to cytotoxic NK cells, a number of non-cytotoxic, so-called helper ILCs, have been identified, initially in the mouse [2–7] and later also in humans [8–11]. In contrast to NK cells, helper ILCs express the α-chain of the IL-7 receptor and depend on IL-7 for development [1]. An increasing body of literature now shows that helper ILCs are important players in tissue homeostasis and inflammation [12]. Similar to T helper cells, ILCs are classified on the basis of expression of master transcription factors and effector cytokines, with group 1 ILCs (ILC1) relying on the transcription factor TBET for production of IFN-γ, ILC2 on GATA3 for production of IL-5 and IL-13, and ILC3 on retinoid-related orphan receptor γt (RORγt) and aryl hydrocarbon receptor (AHR) for production of IL-17 and IL-22 [1]. As the transcription factor and effector cytokine profiles of ILC subsets mirror those of T cell subsets, ILCs have been suggested to be the innate equivalents of T cells [1]. In recent years, it has become increasingly clear that human ILCs are highly plastic cells that, depending on the microenvironment, i.e., inflammation, alter their phenotype and function to meet prevailing needs. However, whether helper ILCs are predominately tissue-resident cells with local self-renewing capacity, or migratory effector cells remains a topic of debate [13, 14].

In this review, we will particularly focus on what is known about human helper ILCs in terms of development, subset heterogeneity, and function. We will also describe the plastic features of human ILCs. Finally, we will report on what is currently known about the interaction between helper ILCs and other lymphocytes, as well as the complementary and redundant features of ILCs and T cells. Although most of these studies have been performed in the mouse, we will highlight how this could be relevant for human homeostasis and disease.

## ILC1

Group 1 innate lymphoid cells (ILC1) comprise both cytotoxic NK cells and non-cytotoxic helper ILC1. ILC1 are commonly defined as IFN-γ-producing cells [1], which depend on the transcription factor TBET (encoded by *Tbx21*) for their development and function whereas NK cells additionally express EOMES [1, 15]. The unique features and heterogeneity of tissue NK cells were recently excellently reviewed elsewhere [16] so for this review, we will focus on helper ILC1. Similarly to Th1 cells, human helper ILC1 are activated by the cytokines IL-12 and IL-18, triggering the release of IFN-γ and TNF [9, 17]. Interestingly, helper ILC1, defined as Lin^−^CD127^+^ cells lacking markers of ILC2 and ILC3, including CD117, CRTH2, and NKp44, were shown to be enriched in inflamed mucosa of patients with Crohn’s disease [9, 17]. In the mouse, helper ILC1 contribute with protection against both intracellular pathogens such as *Salmonella enterica* [18] and *Toxoplasma gondii* [2] and bacteria such as *Helicobacter thyphlonius* [19].

The existence of a defined population of helper ILC1 in the human has recently been a topic of debate [20–22]. Mass cytometry (CyTOF) analysis of around 30 surface markers in several human organs failed to discern a distinct population of IFN-γ-/TBET-producing ILCs using *t-SNE*-based clustering analysis [20]. However, biaxial gating of the same data set could indeed reveal a population of TBET-expressing ILC1 [21], which are likely the ones previously identified in Crohn’s disease intestine [9]. Adding to the helper ILC1 confusion is the observation that cells within the ILC1 population express surface proteins typically expressed by T cells, including CD4, CD5, CD8, and CD28 and several transcripts of TCR and CD3 [23, 24], albeit absent on the cell surface. Whether this is a reflection of the close developmental relationship between ILCs and T cells or represents contamination by T cells remains unclear. The former is supported by the observation that ILC1 do not express surface CD3 or TCR when cultured under T cell promoting conditions [9]. In summary, the human Lin^−^CD127^+^CD117^−^CRTH2^−^NKp44^−^ population, which is commonly referred to as helper ILC1, is likely a heterogenous mix of yet undefined cells and true helper ILC1 with the capacity to express TBET and produce IFN-γ.

## ILC2

Group 2 ILCs were first characterized as IL-13-producing innate lymphocytes in a number of different tissues in the mouse [5–7]. They were later also discovered in human intestinal and nasal tissue, peripheral blood [8], adipose tissue [25], and lung [11] as cells depending on GATA3 [26] and expressing the prostaglandin D_2_ receptor CRTH2 and CD161 [8]. In the mouse, ILC2 additionally rely on retinoic acid receptor (RAR)-related orphan receptor α (RORα) but this remains unclear in humans [26]. Human ILC2 are activated by cell surface ligands, including ICOS and NKp30 [27, 28] and soluble factors such as lipid mediators, including PGD_2_ [29–31] and cytokines IL-25, IL-33, and thymic stromal lymphopoietin (TSLP) [26, 32]. Human ILC2 have been shown to produce the typical type 2 cytokines IL-4, IL-5, IL-9, and IL-13 but also IL-6, IL-8, GM-CSF, and in the mouse additionally amphiregulin (AREG) [7, 11, 26, 33].

The main physiological role for ILC2 is likely in immune defense against helminth infections as demonstrated by mouse studies [6, 7]. In addition, a mouse model of influenza virus infection was used to show how ILC2-produced AREG is involved in respiratory tissue repair [11]. However, ILC2 can also act as inducers of inflammation in mouse airways under viral and allergen exposure [32, 34–36] and ILC2 have been shown to be enriched in several human tissues during type 2-mediated inflammation [15]. In patients with chronic rhinosinusitis with nasal polyps (CRSwNP), ILC2-producing IL-4, IL-5, and IL-13 are found to be significantly accumulated as compared to the healthy nasal mucosa [8, 37, 38]. In asthmatic patients, ILC2 are found to be enriched in bronchoalveolar lavage fluid and sputum [39–41]. ILC2 also play a role in skin repair, where they were found to be enriched in repaired skin near a wound compared to healthy skin before wounding [42].

**Fig. 1:**
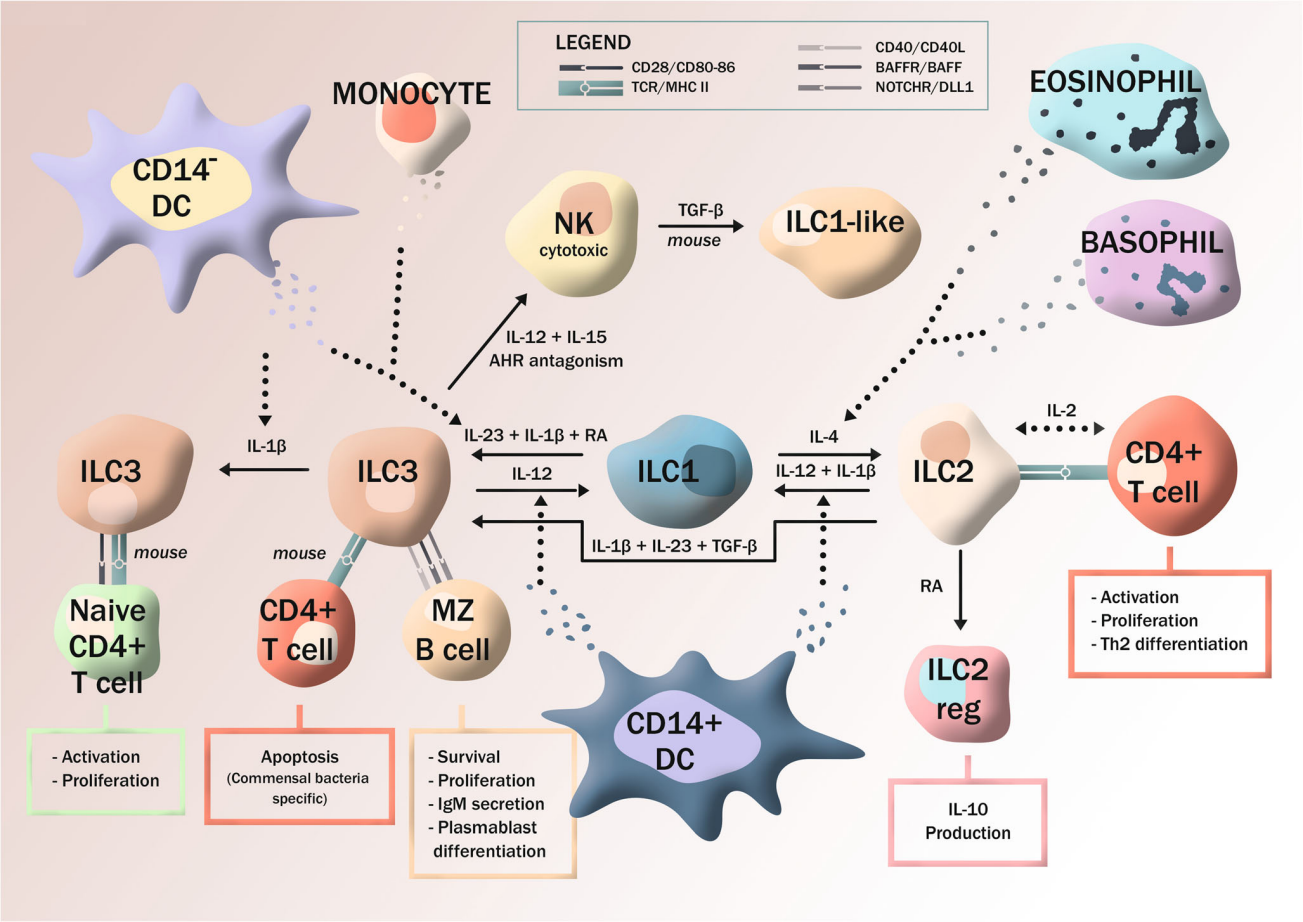
Human ILC plasticity and interaction with other lymphocytes. Human ILCs are highly plastic cells that, depending on the tissue environment, may adapt their function to meet prevailing needs. Human ILC3 can take on features of both cytotoxic NK cells and helper ILC1 under the influence of IL-12, produced by CD14^+^ DCs, with AHR-antagonism acting as an important switch in inducing NK cell function. Recent studies in mice show that TGF-β causes NK cells to convert to ILC1-like cells with reduced cytotoxic capacity. Human ILC1 may revert back to ILC3 in the presence of IL-23, IL-β, and RA, the former produced by subsets of DCs and monocytes. In the mouse, ILC3 suppress commensal-specific Th17 cells in the intestine, whereas ILC3 stimulated by IL-1β have the capacity to activate CD4^+^ T cells. In both humans and mice, ILC3 activate marginal zone (MZ) B cells. Human ILC2 show plasticity towards ILC1 under the influence of IL-1β plus IL-12, a process which can be reverted by IL-4, provided by basophils and eosinophils. Unpublished data indicate that human ILC2 can take on ILC3 functions if exposed to IL-23, IL-β, and TGF-β (Golebski et al.). Additional unpublished data (Morita et al.) reveal that RA induces a regulatory phenotype in human ILC2 (ILC2reg), causing IL-10 release and suppression of CD4^+^ T cell and ILC2 activity. In the absence of RA, ILC2 and CD4^+^ T cells display bidirectional activation via MHCII-TCR interactions

In the mouse, two functionally and phenotypically distinct subsets of ILC2 have been described [43]. Natural (n)ILC2 were described as a homeostatic, lung-residing, and IL-33-responsive cell whereas so-called inflammatory (i)ILC2, activated mainly by IL-25, arose upon inflammation. In the human, there are still no published evidence for existence of ILC2 subsets and single-cell RNA sequencing failed to detect transcriptionally distinct subpopulations of human tonsillar ILC2 [23]. However, such heterogeneity might only be detectable upon type 2 inflammation. Indeed, work from Cezmi Akdis’ group (Swiss Institute of Allergy and Asthma Research, Davos, Switzerland) demonstrates the existence of a regulatory ILC2 population in CRSwNP. These ILC2reg develop under the influence of retinoic acid (RA) express IL-10 and CTLA-4 and suppress the activity of CD4^+^ T cells and ILC2 (Morita et al., personal communication) (Fig. 1). These data nicely parallel similar findings in the mouse [44].

## ILC3

In addition to being Lin^−^CD127^+^, human group 3 ILCs are defined by expression of CD117 and the transcription factors RORγt and AHR [1, 45]. ILC3 are typically activated by IL-1β and IL-23 to produce the effector cytokines IL-22, and to a lesser extent, IL-17A and IL-17F. In the human, these cytokines are produced by distinct subpopulations of ILC3, with IL-22 being produced by ILC3 expressing NKp44 [23, 46, 47], and IL-17A/F by NKp44^−^ ILC3 [23, 47]. Of note, human tonsil ILC3 produce IL-17F with little or no IL-17A, whereas human fetal ILC3 produce substantial amounts of IL-17A [48]. Additionally, human ILC3 produce high levels of TNF, GM-CSF [23, 47].

Lymphoid tissue inducer (LTI) cells were the first members of the ILC3 group, initially reported in 1997 [4]. LTI cells play a role in the organogenesis of secondary lymphoid tissues in the fetus and produce IL-22, IL-17A, and IL-17F [49] as a result of expression of RORyt [3]. In the mouse, LTI cells follow a different developmental path as compared to helper ILC3, since they branch from a section of the common helper-like ILC progenitor (CHILP) that does not express promyelocytic leukemia zinc-finger (PLZF) protein [50]. In humans, it has been difficult to identify a distinct LTI population. However, a recent report showed that ILC3 expressing neurophilin 1 (NRP1) are predominantly found near high endothelial venules in lymphoid but not mucosal tissues [51]. NRP1^+^ ILC3 induce ICAM-1 and VCAM-1 expression on mesenchymal stromal cells, indicative of LTI function. As in the mouse, human NRP1^+^ ILC3 produce significant levels of IL-22 and IL-17A [51]. Hence, these cells might represent the human LTI. It remains however unknown if these cells are developmentally distinct from helper ILC3, as in the mouse.

In addition to the NKp44^+^ and NRP1^+^ ILC3 described above, two other subsets of human ILC3 were initially identified through single-cell RNA sequencing of human tonsil and subsequently confirmed by studies on the protein level [23]. HLA-DR^+^ ILC3 make up around 10% of tonsillar ILC3. Similar to splenic ILC3 in the mouse [52], our group has demonstrated that these cells can take up and process antigens and activate T cells during conditions of inflammasome activation (Rao et al., unpublished observation). The actual physiological relevance of this observation remains however to be determined.

Finally, through single-cell RNA sequencing of human tonsils, we identified a subpopulation of CD62L^+^ ILC3. These cells co-express CD45RA and show an inability to release IL-22 and IL-17 in response to IL-1β and IL-23. It is therefore tempting to speculate that these cells are naïve ILC3, capable of differentiating to mature phenotypes as recently described for peripheral blood ILC3-like ILC precursors [53]. The developmental and functional relationship between ILC precursors in blood and tonsillar CD62L^+^ ILC3 remains unexplored.

In mice, ILC3 have been shown to have both pathological and protective roles. One of the main effects of ILC3 in the gut is maintenance of barrier integrity through production of IL-22 [54, 55]. Prevention of bacterial translocation [56] and pathogen clearance was also reported in mice infected with *Candida albicans* [57], *Clostridium difficile* [58], *Salmonella typhimurium* [59], *Streptococcus pneumonia* [60], rotavirus [61], and intestinal helminthes [62]. ILC3 play a protective role during *Citrobacter rodentium* and *Escherichia coli* infection, where ILC3-derived IL-22 is needed for survival in immunocompromised mice, while providing protection during the first phase of infection in immunocompetent mice [55, 63, 64]. However, ILC3 also contribute to pathology. In *Salmonella enterica*-infected mice, ILC3 produce inflammatory cytokines such as IL-17 and IFN-γ, exacerbating the infection [65]. IL-17 production also linked to the development of obesity-associated asthma [66]. An excessive IL-22 production in the gut was also linked to the formation of tumors [67].

In humans, the dominating ILC3 population in the gut is IL-22-producing NKp44^+^ ILC3 [47], which likely, as judged from mouse studies, plays a role in intestinal homeostasis. Supporting this, appearance of IL-22-producing, gut-homing NKp44^+^ ILC3 in the blood of acute myeloid leukemia (AML) patients following conditioning treatment and hematopoetic stem cell transplantation (HSCT) is associated with protection against development of graft-versus-host disease (GVHD) [68]. In contrast, in gut pathology, IL-17-producing ILC3, mainly those lacking NKp44, are enriched in the inflamed gut of patients affected by Crohn’s disease [69]. In contrast to the gut, accumulation of NKp44^+^ ILC3 in the skin is associated with pathology. In patients with psoriasis, IL-22-producing NKp44^+^ ILC3 are accumulated in the blood and skin and their frequencies correlate with disease severity [70, 71]. Hence, depending on the tissue context, human NKp44^+^ ILC3 may play protective or disease-causing roles.

## Development of human ILCs

Whereas the ontogeny of ILCs in the mouse is being unraveled using advanced genetic engineering [72], the understanding of human ILC development is far more limited. Human ILCs can be derived from adult CD34^+^ bone marrow precursors as demonstrated by in vitro experiments [73]. These data are supported by the fact that hematopoetic stem cell transplantation causes reconstitution of donor ILCs in patients [68] and in a humanized mouse model [53]. Furthermore, two groups reported on a more defined CD34^+^ precursor residing in secondary lymphoid tissues, expressing CD117, CD45RA and RORyt that could give rise to ILCs, but not T cells [74, 75]. One group showed that this cell population contained progenitors of all ILC populations, including NK cells [74], whereas the other only demonstrated ILC3 precursor activity [75]. Recently, a human CD34^−^ ILC precursor circulating in peripheral blood was described as a cell expressing CD7, CD127, and CD117 but lacking lineage markers and transcription factors associated with mature ILCs, including RORyt [53]. This population was composed of both uni- and multipotent precursors of all ILC populations, as demonstrated by both in vitro and in vivo experiments. Although it is tempting to speculate that CD34^+^ multipotent ILC precursors [74] give rise to CD34^−^ multipotent and then unipotent ILC precursors, this remains to be formally proven. It also remains unknown what regulates the organ seeding of this systemic CD34^−^ ILC precursor and which conditions dictate the local ILC development from these precursors in various tissues.

## Human ILC plasticity

Human ILCs display significant plasticity, defined as the capacity of a mature ILC population to acquire the features associated with another mature ILC population. This dynamic feature of human ILCs might be an efficient way of rapidly adapting immunity to prevailing conditions in tissues without recruitment of cells from other tissue sources. The mechanisms underlying ILC plasticity obviously serve as attractive therapeutic targets.

### ILC3-ILC1 plasticity

Although the total frequency of ILCs remains unaltered in the inflamed lamina propria of Crohn’s disease patients as compared to controls, the frequency of ILC1 increases in Crohn’s disease, making ILC3–ILC1 conversion an attractive mechanism for ILC1 enrichment [9, 17]. Indeed, it was demonstrated that human ILC3 can differentiate towards ILC1 under the influence of IL-12, released by CD14^+^ DCs in the inflammatory intestinal mucosa (Fig. 1). In this plastic process, ILC3 loose the transcription factor RORγt and acquire TBET, upregulating IFN-γ while losing IL-22 production [9]. This conversion is reversible, since ex-ILC3 can be re-differentiated to the ILC3 phenotype if cultured with IL-2, IL-23, and IL-1β, with RA enhancing this mechanism [17]. Also, freshly isolated helper ILC1 are capable of converting towards an ILC3 phenotype and IL-22 production whereas NK cells lack this ability [17].

In the mouse, IL-12 has been shown to cause differentiation of NKp46^+^ ILC3 towards an IFN-γ-producing, TBET-expressing ILC1-like cell [18]. As shown in humans, differentiated mouse NKp46^+^ ILC3 had the ability to convert back to their original RORyt^+^ profile under the influence of IL-23.

### ILC2-ILC1 plasticity

Similar to what was reported on ILC3–ILC1 plasticity in the intestine, human ILC2 have been shown to display plasticity towards ILC1 during type 1 inflammatory conditions (Fig. 1), such as Crohn’s disease and chronic obstructive pulmonary disease (COPD) [37, 76–78]. As expected, the main cytokine needed for this conversion is IL-12. Additionally, IL-1β is needed as it primes the ILC2 to express Th1 cell-related genes like *tbx21* (encoding TBET) and upregulate IL12Rβ2 receptor expression, making the ILC2 receptive to IL-12. The cooperation between IL-1β and IL-12 changes the epigenetic state by activating the *IFNG* promoter [78], inducing differentiation of ILC2 to a GATA3-/TBET-expressing cell that produces IFN-γ. The conversion of ILC2 to “ex-ILC2” can be inhibited and reversed by IL-4 [37], a cytokine that maintains ILC2 phenotype and functions by boosting GATA3 and CRTH2 expression.

### ILC2-ILC3 plasticity

In the mouse, a particular subset of ILC2, iILC2, was shown to express high levels of GATA3 but also intermediate levels of RORyt [43, 79]. In vivo experiments of transferred iILC2 in *C*. *albicans*-infected mice showed how iILC2 lost IL-13-producing capability while becoming IL-17 producers, resembling an ILC3-like cell [43].

In humans, there are still no published studies on ILC2–ILC3 plasticity, but there are studies underway from Hergen Spits’ lab (Academic Medical Center, Amsterdam, the Netherlands) demonstrating how human ILC2 in the presence of IL-1β, IL-23, and TGF-β become IL-17 producers (Fig. 1) (Golebski et al., personal communication). Such cells could be important for recruitment of neutrophils to type 2 inflamed tissues.

### ILC3-NK cell plasticity

Studies in the mouse suggest that helper ILCs and NK cells follow distinct paths of development [50, 80]. Helper ILCs are thought to arise from a common helper innate lymphoid cell progenitor (CHILP) to then differentiate into TBET^+^ ILC1, GATA3^+^ ILC2, and RORγt^+^ ILC3, while NK cells develop from a distinct NK cell progenitor (NKp) and have no history of RORγt expression [81]. As described above, this might be different in humans as it has been shown that NK cells and all helper ILC subsets can be derived from CD34^+^RORγt^+^ precursors [82] and from circulating ILC3-like multipotent precursors [53]. Additionally, recent reports hint towards the possibility of fully mature RORγt^+^ ILC3 showing plasticity towards NK cells. One report described how human ILC3 from pediatric tonsils and from humanized mouse tissues have the ability to differentiate to cells displaying many characteristics of early-differentiated (stage 4) NK cells [83] (Fig. 1). The key cytokines promoting this phenotypical change are IL-12 and IL-15, which upregulate the NK cell transcription factor EOMES, along with other NK cell surface markers, like CD94, NKG2A, CD56, and CD16. These NK-like cells produce type 1 cytokines IFN-γ and TNF, but also have the machinery to create and release cytotoxic granules, thus being able to kill tumor target cells. Interestingly, AHR has been described as the transcription factor responsible for preventing IL-22-producing ILC3 to differentiate into stage 4 NK cells [82]. IL-1β induces AHR, but also several features of ILC1, including IL12Rβ2 receptor. Hence, it becomes obvious that ILC plasticity is dictated by the tissue microenvironment. So far, no actual physiological situation where ILC3–NK cell plasticity occurs in humans has been described and although there are indications from mouse studies [84], there are no clear evidence of this actually occurring in vivo so far. It is however tempting to speculate that disturbances in dietary AHR uptake in the gut might promote ILC3–NK cell plasticity, causing decreased IL-22-mediated barrier integrity and increased NK cell-mediated inflammation. This remains however to be determined.

### NK cell-ILC1 plasticity

In the mouse, conversion from NK cells to less cytotoxic, ILC1-like tissue-resident NK cells was recently suggested to be driven by TGF-β [85] (Fig. 1). This conversion was attributed to the strict regulation of the signal transducer SMAD4 [86]. This phenotypical switch was described in a tumor context, where NK cells differentiating into ILC1-like cells lost the ability to control tumor or viral burden. These reports point towards the possibility that ILC1 in a tumor environment are NK cells that have undergone differentiation, lost their cytotoxicity, and upregulated transcription factors typically associated with tissue resident but not conventional cytotoxic NK cells, like Hobit and TIGIT. TGF-β also induces features of tissue-resident NK cells in humans and it is likely that human CD49^+^ tissue-resident NK cells [87] develop under the influence of TGF-β. However, whereas human CD49^+^ NK cells are EOMES^dim^TBET^+^, similarly to helper ILC1, they still express KIR and NKG2C, indicating that they are NK cells rather than helper ILC1. Hence, it still remains unclear if NK cells can acquire all features of helper ILC1, rather than converting to NK cells with a tissue-resident phenotype.

## Interaction with other cell types

Through their ability to advance the cascade of inflammatory reactions, ILCs are involved in a myriad of interaction with other immune cells. Myeloid cells are able to sense danger signals originating from invading pathogens or damaged tissue and secrete cytokines that consequently instruct ILCs [88]. Cytokine networks involved in the cross-talk between ILCs and myeloid cells have been recently extensively reviewed by Mortha and Burrows [88]. Thus, in the present review, we will summarize the current understanding of how ILCs interact with other cells of lymphoid origin.

### The interplay between ILC2 and Th2 cells

Mouse studies have demonstrated that ILC2 primes DCs to induce Th2 differentiation [89, 90]. However, ILC2 also seem capable of directly influencing T cell responses. Several studies of mouse models have demonstrated the ability of ILC2 to drive Th2 responses either directly through MHCII-TCR interactions [91, 92], or by providing signals for Th2 polarization and reciprocally enhancing type 2 immune responses [91–94] (Fig. 1). Murine ILC2 were shown to exhibit APC-like functions and to present antigens to T cells, leading to their proliferation and driving MHCII-independent, but contact-dependent Th2 polarization. In turn, T cell-derived IL-2 enhanced ILC2 cytokine production and induced their proliferation [91]. Furthermore, MHCII-mediated cross-talk was shown to potentiate IL-13 production by ILC2 in addition to being indispensable for *Nippostrongylus brasiliensis* expulsion [92]. The potential of murine ILC2 to drive Th2 responses was attributed to IL-4 secretion and expression of the co-stimulatory molecule OX40L [93]. More recently, PD-L1-expressing ILC2 were shown to promote early Th2 polarization and IL-13 production while accelerating anti-helminth responses in vivo [95]. Nonetheless, the role of ILC2 in priming T cell responses might be strictly dependent on the route of infection, since systemic antigen delivery initiates Th2-driven lung inflammation, independent of ILC2 [94].

Human ILC2 have also been implicated in antigen presentation. Peripheral blood-derived ILC2 expanded with 100 U/ml of IL-2 and gamma-irradiated feeder cells expressed HLA-DR and induced antigen-specific cytokine responses in house dust mite allergen-specific T cell lines [92]. However, the role of ILC-dependent antigen presentation in human allergic inflammation remains to be elucidated.

Besides interacting with Th2 cells, IL-9^+^ ILC2 were recently shown to promote the activity of Tregs in mice by expressing ICOSL and GITRL [96]. Supporting a role for IL-9^+^ ILC2 in resolution of inflammation in humans, rheumatoid arthritis patients in remission exhibited higher frequencies of IL-9^+^ ILC2 in both blood and synovial tissue as compared to patients with active inflammation.

### The interplay between ILC3 and adaptive lymphocytes

The predominant ILC population in the human intestine is ILC3 but there are still no evidence for ILC3-T cell interaction playing a role in gut homeostasis or inflammation in humans. Interestingly, in the murine intestine, MHCII^+^ ILC3 have been shown to suppress T cell responses while promoting immune tolerance to commensal bacteria [97, 98] (Fig. 1). Reduction of such MHCII^+^ ILC3 perpetrated colitis in mice and reduced frequency of HLA-DR^+^ ILC3 was associated with early-onset IBD in pediatric patients. However, in another murine study, it was demonstrated that IL-1β stimulation leads to the activation of peripheral ILC3, marked by MHCII upregulation and expression of T cell co-stimulatory molecules [99] (Fig. 1). MHCII^+^ ILC3s primed CD4^+^ T cell responses in vitro and in vivo. These studies demonstrate that antigen presentation by ILCs and its effects on T cells are strongly dependent on the tissue localization and are shaped by their immediate microenvironment. One important difference between mouse and human, which might influence antigen-specific ILC-T cell interactions, is that just like ILCs, activated human T cells are able to express HLA-DR and thus, might participate in antigen presentation. Whether such an expression is contributing to a mutual redundancy, or HLA-DR-expressing ILCs and T cells are involved in different physiological/pathological processes in humans remains unknown.

Reciprocal inhibition of intestinal T cells and ILC3 was described in mice, where elevated ILC numbers and increased IL-22 expression as well as antimicrobial peptide production were observed in the absence of intestinal CD4^+^ T cells [100]. More recently, Mao et al. [101] unveiled the underlying mechanism of sequential innate and adaptive lymphocyte-dependent control of the gut microbiota during development in mice. In the early phase of weaning, concomitant with the expansion of segmented filamentous bacteria (SFB), CCR2^+^ monocyte/mDC-derived IL-23 triggers IL-22 production by intestinal ILC3, which in turn induces AMP production by intestinal epithelial cells. With the expansion and maturation of the adaptive immune system, T_reg_ and T_H_17 cells suppress IL-23 production by monocytes and reduce SFB abundance, respectively. This leads to suppression of the ILC3-mediated microbiota control axis. In the absence of T cells, IL-22 production by ILC3 persists, resulting in impaired lipid metabolism in the small intestine of mice [101].

ILCs have been shown to regulate B cell responses in mice [102, 103] and humans [104, 105] (Fig. 1). Murine RORγt^+^ ILCs aid the regulation of IgA production in the lamina propria via two distinct pathways involving expression of either soluble or membrane-bound lymphotoxin (LT). While membrane-bound LTα_1_β_2_ promotes iNOS expression by DCs and regulates T cell-independent IgA production, soluble LTα3 promotes T cell homing to the lamina propria, which in turn induce IgA [102]. In humans, splenic ILCs localized at the marginal zone were shown to provide help to innate-like B cells via co-stimulatory factor such as BAFF, CD40L, and DLL1 [105]. At the same time, splenic ILCs produce IL-8 and GM-CSF, which recruit and activate neutrophils to further enhance B cell responses. Studies in mouse models further confirmed the role of splenic ILCs in activation of marginal zone B cells. Noteworthy, several phenotypic differences between mice and humans were described, with murine ILCs expressing APRIL and DLL1, but lacking BAFF and CD40L expression [105]. Moreover, TLR-induced CD40L expression has been recently demonstrated in circulating human ILC2, resulting in these cells gaining the potential to promote IgE production by B cells [104].

Adding a further layer of regulation, a recent study in mice suggested an important role of ILC3 in maintaining adult lymph node homeostasis, and in particular regulating T and B cell migration into lymph nodes [106]. The precise mechanism of ILC3-dependent T and B cell trafficking into lymph nodes remains to be elucidated. However, this once again demonstrates how the absence of ILCs and the subsequent cellular cross-talk might disrupt an entire physiological network. This remains however to be elucidate further, especially in the human setting.

## ILC and T cell complementarity

The distinct features of ILCs and T cells allow for complementarity and redundancy between these innate and adaptive immune systems. Whereas T cells are activated through MHC-peptide-TCR interactions and co-stimulatory signals, ILCs characteristically lack expression of rearranged antigen receptors. Instead, these cells are primed by surrounding cytokines, hormones, and lipid mediators and may additionally be susceptible to environmental stimuli [30, 45, 107, 108]. In temporal space, the differing modes of activation allow ILCs to act as first responders, with T cells picking up the pace following activation by antigen-presenting cells and clonal expansion. The ability of ILCs to act as first responders is further compounded by the observation that many tissues, at least in the mouse, harbor resident ILC populations [13], preceding the necessity for these cells to migrate prior to eliciting an immune response. In contrast, the majority of T cells must first acquire expression of homing receptors and migrate from secondary lymphoid organs to the effector site. On a situational level, the complementary localization of these two immune subsets also translates to the capacity for ILCs to respond rapidly and robustly at local sites, whereas T cells may respond both locally and systemically. Lastly, whereas T cells have evolved to respond towards attacks on the immune system, recent publications highlight the capacity for ILCs to additionally respond to more subtle alterations in immune homeostasis. For example, constitutive IL-5 secretion by ILC2 was demonstrated to be influenced by circadian rhythms and food intake [109], whereas IL-22 production by ILC3 is regulated by AHR, a transcription factor responsive to both xenobiotics and organic compounds [45, 110]. Furthermore, lL-13 production by ILC2 was shown to promote tuft cell development [111], and IL-22 expression by ILC3 is known to induce proliferation and survival of epithelial cells [112]. Therefore, it seems reasonable to argue that ILCs and T cells are orchestrated to act in harmony with one other, complementing one another in spatial, temporal, and functional aspects [113]. This has recently been the focus for intensive research, primarily in mice, but also in humans, which we will summarize below.

### Lessons from immune-compromised and immune-competent mice and humans

In 2015, a study by Song et al. addressed the contribution of ILC and T cells to anti-CD40-mediated colitis, making effective use of engineered mice harboring severely reduced numbers of NKp46^+^ ILC3, all ILC3, T cells or both ILC3 and T cells. Interestingly, anti-CD40 colitic mice lacking both T cells and ILC3 displayed milder histopathology than mice lacking T cells only, where ILC3 production of GM-CSF was shown to be required for recruitment of pro-inflammatory monocytes to the site of inflammation [114]. More recently, Brasseit et al. have expanded on these findings by demonstrating that mice which lack ILC3 display milder colitis. Therefore, ILC appear to contribute uniquely to the innate immune response in the anti-CD40-mediated colitis model [115].

In turn, an in-depth study of ILC and T cells in the CD4 T cell transfer colitis model, in which the adaptive immune compartment contributes significantly, has shed a more nuanced light on the contribution of innate and adaptive immune cells to colitis development. Transfer of colitogenic CD4 T cells to *Rag1*^*−/−*^ mice depleted of ILC highlighted that CD4^+^ T cells, but not ILCs, are critical for induction of colitis [115]. Nonetheless, absence of ILC3 exacerbated histopathological signs of colitis, arguing for a non-redundant and time-specific function of ILC3 in the CD4 T cell transfer colitis model [115].

A notable limitation of these and other studies is the examination of ILC function in the context of immune-compromised mice. However, a number of recent publications have successfully dissected the individual contributions of ILC and T cells in specific immune settings. In a model of *C. rodentium* infection developed to examine the contribution of ILC to bacterial infection, ILC were shown to exacerbate pathogenesis of *C. rodentium*-mediated infection in mice lacking T cells. Specifically, lack of non-NKp46^+^ ILC3, but not NKp46^+^ ILC3, accelerated body weight loss and mortality of mice as compared with mice lacking T cells only [114]. These findings were subsequently corroborated by a second study wherein deletion of key ILC3 genes confirmed IL-22 production by NKp46^+^ ILC3 to be redundant for the control of *C. rodentium* infection in the presence of T cells [116]. Importantly, however, NKp46^+^ ILC3 were shown to be essential for cecal homeostasis, where *C. rodentium*-infected mice lacking NKp46^+^ ILC3 presented with a decreased cecum size and histopathological signs of hyperplasia and inflammation.

Another intriguing recent publication has additionally shed new light on the complementarity and redundancy of ILCs and T cells in maintaining the delicate balance between bacterial control and gut homeostasis. Studying intestinal epithelial cells (IEC) and ILC3 activation through pSTAT3 phosphorylation, Mao et al. observed microbiota-dependent pSTAT3 signaling in ILC3 and IEC in mice lacking T cells but not WT mice [101]. Subsequently, a more detailed analysis showed that neonatal mice had neither pSTAT3^+^ ILC3 nor IEC, and appearance of pSTAT3^+^ cells was linked to weaning of mice. Furthermore, as the adaptive immune system evolved, ILC3 in WT mice lost their activated state, whereas activation of ILC3 in adult mice lacking T cells persisted. In this, Tregs were shown to prevent ILC3 activation through suppression of IL-23 production from CCR2^+^ myeloid cells, whereas Th_17_ cells decreased microbial burden and as such indirectly inhibited ILC3 activation. In addition, activated ILC3 and T cells differentially regulated segmented filamentous bacteria (SFB) in the small intestine, where ILC3 prohibited the development of SFB into long filamentous forms and T cells prevented attachment of SFB to IEC. Thus, providing evidence that ILCs carry out complementary and non-redundant functions in the intestine of young and adult mice [101].

In humans, little research has been conducted examining ILC redundancy. However, one recent study by Vely et al. showed that severe combined immunodeficiency (SCID) patients with mutations in the genes *IL2RG* and *JAK3* are deficient in circulating helper ILCs and NK cells [113]. This can be explained by the requirement for IL-7 and IL-15 signaling in the survival of these cells, where IL-7 and IL-15 signals are integrated by the common γc cytokine receptor and the downstream JAK3 tyrosine kinase. Interestingly, ILCs and NK cells are not properly reconstituted after bone marrow transplantation, although their T and B cell pools are replenished [117]. Alongside, the authors could detect little or no tissue-resident CD3^−^ NKp46^+^ or CD3^−^ CD11b^−^ ICOS^+^ ILC as examined through staining of skin and gut paraffin-embedded and frozen tissue sections. A subsequent study of the long-term medical history of these *IL2RG-* and *JAK3*-deficient SCID patients demonstrated no significant increased risk for a number of major medical afflictions, such as HPV, respiratory infections, or disease as compared with control patients. Taken together, this data argues for some level of ILC redundancy in humans. Nonetheless, as staining of skin and gut tissue sections could not exclude the presence of all tissue-resident ILCs, and both patient cohort size and the number of medical conditions examined were limited, caution should be taken when drawing conclusions. This is particularly true when considering the large number of studies that have shown that ILCs are major sources of effector cytokines in human disease [9].

Overall, it seems reasonable to conclude that most ILC populations carry out unique functions, whereby the impact of ILC function should be viewed in the context of the stage of immune development, immune competency, and localization of the immune response. The existing literature also demonstrates the need for continued studies of human subjects, to ultimately determine the complementarity and redundancy of human ILCs.

## Conclusion

Since the discovery of the ILC family, research in the area has shown that these highly plastic cells display functions that can be both complementary and redundant within the immune system. In the human setting, ILC undoubtedly serve as significant sources of cytokines that drives pathology. Additionally, by acting in a network of other immune cells, ILC can propagate their functions beyond those directly mediated by surface ligands and secreted effector cytokines. There are a number of urgent questions that deserve attention in the field. One such relates to the relative importance of local proliferation and plasticity, development from local precursors, and recruitment of mature ILCs and undifferentiated precursors to the total functional pool of ILCs in a given tissue. It also remains obscure how and where human ILCs develop. Other questions deal with the confusing field of ILC1. What is the developmental and functional relationship between helper ILC1, TGF-β-induced ILC1, and tissue-resident NK cells, which all share common features? Furthermore, which cells make up the CD117^−^ fraction of ILCs that we call helper ILC1 in humans?

And of course, ultimately, we need to understand how to specifically target ILCs for treatment of acute and chronic inflammatory diseases, to which subsets of ILCs have been shown to contribute.
